# Revisiting rhinoscleroma microbiology: a case with concordant dual-specimen bacterial isolation

**DOI:** 10.3389/fcimb.2026.1837785

**Published:** 2026-08-21

**Authors:** Lorenzo Drago, Nikolaos Papanikolau, Andrea Preti, Francesco Mozzanica, Luigi Regenburgh De La Motte, Giuseppe Pelosi, Loredana Deflorio

**Affiliations:** 1 Unità Operativa Complessa (UOC) Laboratory of Clinical Medicine with Specialized Areas, Istituto di Ricovero e Cura a Carattere Scientifico (IRCCS) MultiMedica, Milan, Italy; 2 Department of Biomedical Health Sciences, University of Milan, Milan, Italy; 3 Inter-Hospital Pathology Division, Istituto di Ricovero e Cura a Carattere Scientifico (IRCCS) MultiMedica, Milan, Italy; 4 Department of Otorhinolaryngology, Istituto di Ricovero e Cura a Carattere Scientifico (IRCCS) Multimedica, Milan, Italy; 5 Department of Clinical Sciences and Community Health, University of Milan, Milan, Italy; 6 Department of Oncology and Hemato-Oncology, University of Milan, Milan, Italy

**Keywords:** antimicrobial resistance, deep-tissue culture, dual-specimen analysis, Morganella morganii, Rhinoscleroma

## Abstract

**Background:**

Rhinoscleroma is a chronic granulomatous infection of the upper airway traditionally considered a monomicrobial disease caused by *Klebsiella pneumoniae* subsp. *rhinoscleromatis*. However, limited use of deep-tissue microbiological sampling may have contributed to underrecognition of additional bacterial isolates and microbial complexity.

**Methods:**

We report a biopsy-confirmed case of nasal rhinoscleroma in a previously healthy adult. Histopathological evaluation and microbiological analysis were performed on deep intranasal tissue. Bacterial identification was conducted using VITEK 2 and MALDI-TOF mass spectrometry. Antimicrobial susceptibility testing was interpreted according to EUCAST 2025 criteria.

**Results:**

Histology demonstrated characteristic Mikulicz cells. Microbiological analysis revealed *K. pneumoniae* (consistent with subsp. *rhinoscleromatis*) together with *Morganella morganii* from deep-tissue biopsy. Importantly, both organisms were also recovered from an independent nasal swab specimen, with concordant antimicrobial susceptibility profiles. *M. morganii* showed resistance to aminopenicillins and amoxicillin–clavulanate while remaining susceptible to piperacillin–tazobactam, consistent with its intrinsic AmpC β-lactamase profile. Microbiological findings prompted targeted modification of antimicrobial therapy, and targeted therapy with oral levofloxacin was initiated.

**Conclusion:**

This case suggests possible microbial complexity in rhinoscleroma and highlights the value of deep-tissue and concordant multi-sample microbiological evaluation. Recognition of co-isolated organisms may have implications for antimicrobial selection and stewardship, although their pathogenic contribution requires further investigation.

## Introduction

Rhinoscleroma is a chronic granulomatous infection of the upper respiratory tract caused by *Klebsiella pneumoniae* subsp. *rhinoscleromatis*, a Gram-negative encapsulated bacillus with marked tropism for the nasal mucosa and adjacent structures (Wf, 2000; Mukara et al., 2014; Umphress and Raparia, 2018). Although now rare in industrialized countries, it remains endemic in parts of Africa, the Middle East, South America, and Eastern Europe, where delayed diagnosis is common due to its indolent progression and tumor-like presentation (Wf, 2000; Mukara et al., 2014; Umphress and Raparia, 2018). Histopathologically, the disease is characterized by Mikulicz cells—large vacuolated macrophages containing intracellular bacilli—which represent the diagnostic hallmark (Wf, 2000; Mukara et al., 2014; Umphress and Raparia, 2018).

Traditionally, rhinoscleroma has been regarded as a strictly monomicrobial infection. However, this concept is largely based on earlier microbiological investigations relying on superficial sampling or conventional culture techniques. Recent applications of deep-tissue microbiological assessment suggests that additional bacterial species may occasionally be involved, particularly in chronic inflammatory conditions of the upper airway, with potential implications for diagnosis and treatment.

*Morganella morganii* is an opportunistic Gram-negative pathogen increasingly recognized in polymicrobial infections and characterized by intrinsic chromosomal AmpC β-lactamase production, conferring resistance to aminopenicillins and amoxicillin–clavulanate (Jacoby, 2009; Liu et al., 2016; Tamma et al., 2019). Failure to recognize this resistance mechanism may lead to inappropriate empirical therapy.

Here, we report a biopsy-confirmed case of nasal rhinoscleroma in which deep-tissue microbiological analysis suggested possible polymicrobial involvement, highlighting the diagnostic value of comprehensive sampling and its implications for antimicrobial stewardship.

## Methods

This case report was prepared in accordance with the CARE (CAse REport) guidelines. The study describes a biopsy-confirmed case of nasal rhinoscleroma in a previously healthy adult. Deep intranasal tissue samples were obtained during endoscopic biopsy and processed for both histopathological and microbiological analyses. Histopathological evaluation included hematoxylin–eosin (H&E; Bio-Optica, Milan, Italy) and periodic acid–Schiff (PAS; Bio-Optica, Milan, Italy) staining to identify characteristic intracellular bacilli within Mikulicz cells. For microbiological analysis, the biopsy specimen was collected in Tryptic Soy Broth (TSB; bioMérieux, Marcy-l’Étoile, France), which served as the transport medium, and processed upon arrival in the laboratory. The sample was vortexed to improve specimen homogenization prior to culture and inoculated onto Columbia CNA agar, MacConkey agar, Sabouraud agar, and PVX agar plates (bioMérieux, Marcy-l’Étoile, France) using a four-quadrant streaking technique to obtain isolated colonies. Columbia CNA agar, MacConkey agar, and Sabouraud agar plates were incubated aerobically at 37 °C, whereas PVX agar plates were incubated in a CO_2_-enriched atmosphere at 37 °C. Culture plates were inspected after 24 h and maintained under the same incubation conditions for a total of 48 h before final microbiological interpretation. Bacterial growth was assessed qualitatively based on colony recovery on culture media. No quantitative or semiquantitative colony count was performed as part of routine diagnostic processing. In addition to the biopsy specimen, an independent nasal swab specimen was collected and processed using the same microbiological workflow. *Klebsiella pneumoniae* was recovered from PVX agar, whereas *Morganella morganii* was isolated on MacConkey agar. Bacterial identification was performed using an automated system (VITEK 2; bioMérieux, Marcy-l’Étoile, France) and matrix-assisted laser desorption ionization–time of flight mass spectrometry (MALDI-TOF MS; AUTOF MS 1000, Eurobio Scientific, Les Ulis, France). MALDI-TOF confirmed identification at the species level, while attribution to *K. pneumoniae* subsp. *rhinoscleromatis* was based on biochemical identification integrated with the characteristic clinical presentation and histopathological findings. Antimicrobial susceptibility testing was performed using VITEK 2 and interpreted according to the European Committee on Antimicrobial Susceptibility Testing (EUCAST 2025) criteria. Minimum inhibitory concentrations (MICs) were recorded for all tested antimicrobial agents.

## Results

**Figure 1:**
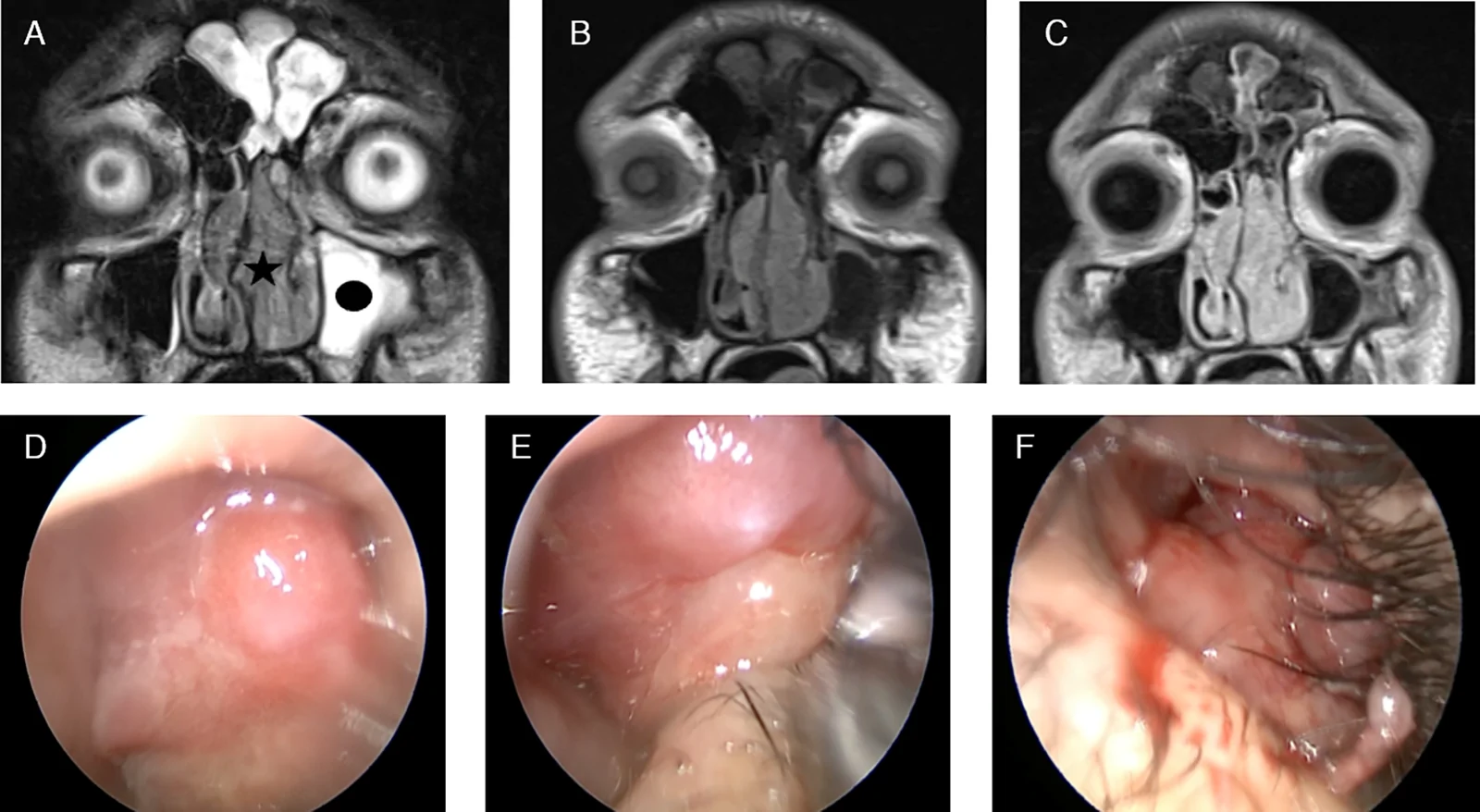
Radiologic and endoscopic findings in nasal rhinoscleroma: **(A–C)** Axial magnetic resonance imaging (MRI) scans (T2-weighted in A, T1-weighted in B, and contrast enhanced T1-weighted in C) of the nasal cavity and paranasal sinuses showing an infiltrative soft-tissue lesion involving the nasal septum and adjacent nasal structures, consistent with a chronic granulomatous process, without evidence of bone destruction. **(D–F)** Endoscopic views of the nasal cavity demonstrating an irregular, polypoid mucosal mass with friable surface and inflammatory appearance.

**Figure 2:**
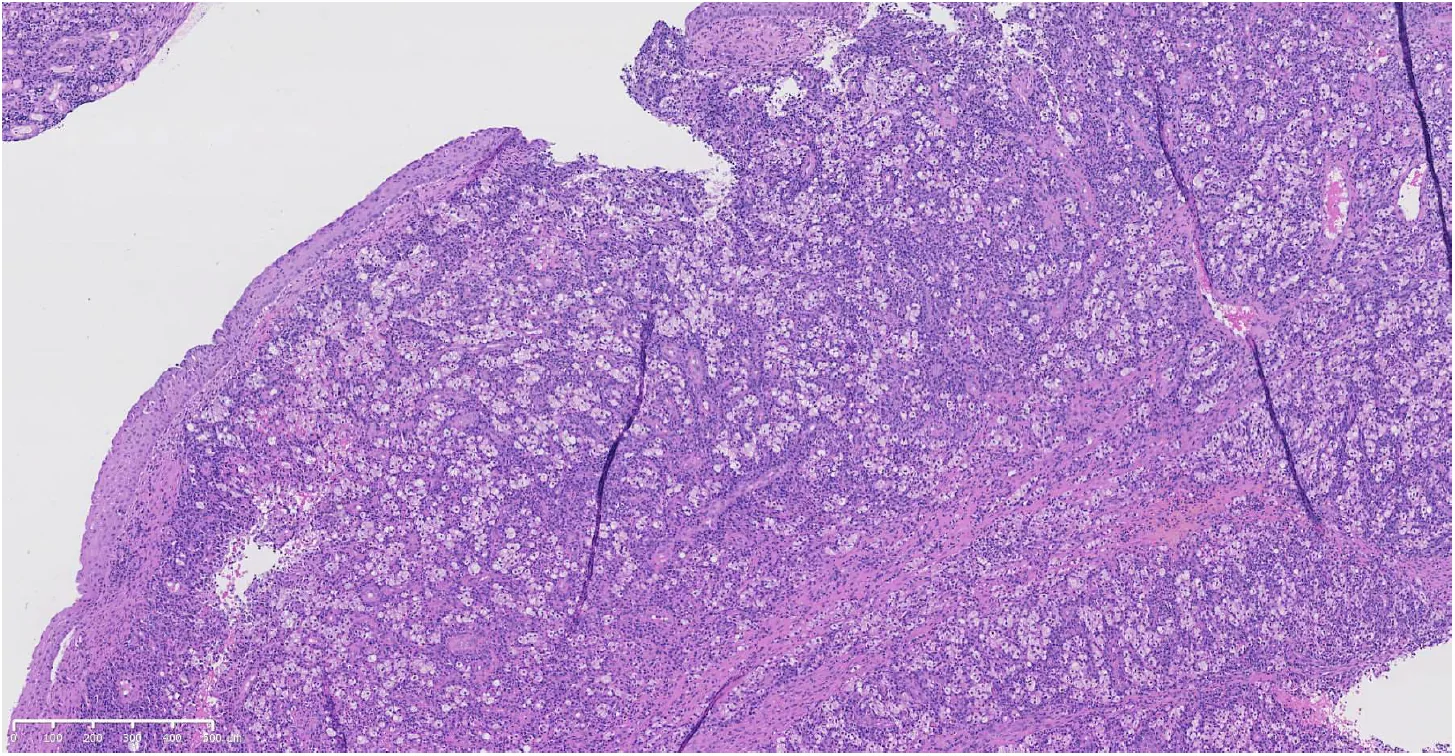
Histopathologic findings in rhinoscleroma showing granulomatous inflammation with numerous vacuolated macrophages (Mikulicz cells).

**Figure 3:**
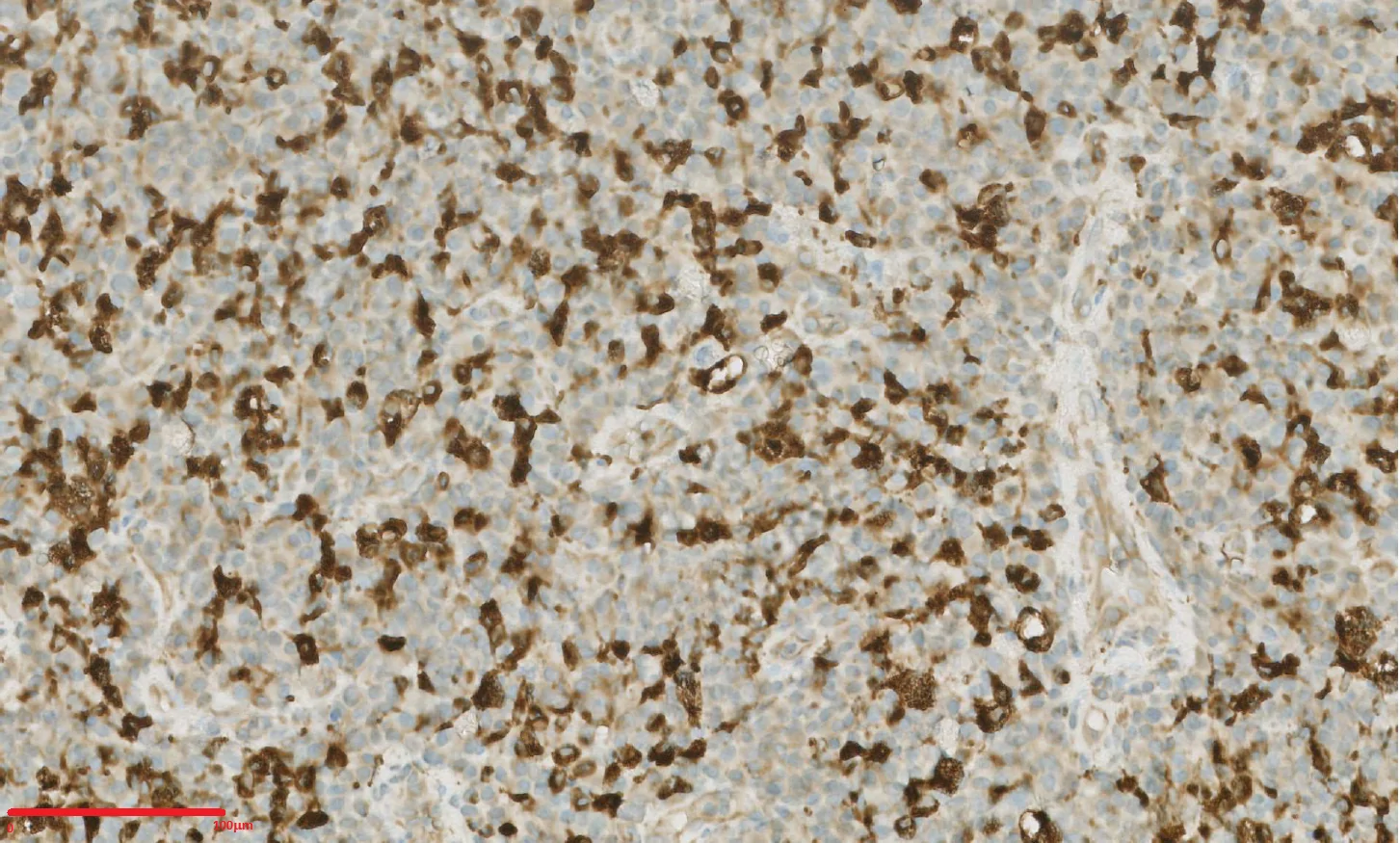
Immunohistochemical staining (CD68) highlighting macrophage-rich inflammatory infiltrates.

A previously healthy young adult male of Egyptian origin presented with a history of progressive nasal obstruction, intermittent epistaxis, and persistent mucopurulent discharge lasting more than one year. No systemic symptoms or immunosuppressive conditions were reported. Nasal endoscopy revealed a friable polypoid mass causing marked airway obstruction (**Figures 1D–F**). Magnetic resonance imaging showed an infiltrative soft-tissue lesion involving the nasal septum and adjacent structures without bone destruction, consistent with a chronic inflammatory or granulomatous process (**Figures 1A–C**). Endoscopically guided deep intranasal biopsy was performed to exclude malignancy. Histopathological examination demonstrated dense chronic inflammatory infiltrates with numerous vacuolated macrophages (Mikulicz cells), consistent with rhinoscleroma (**Figure 2**). Periodic acid–Schiff staining confirmed intracellular bacillary structures. Immunohistochemical staining (CD68) supported a macrophage-rich granulomatous pattern (**Figure 3**). Deep-tissue specimens obtained during biopsy were simultaneously processed for microbiological investigation. Culture yielded *K. pneumoniae* (consistent with subsp. *rhinoscleromatis*) together with *M. morganii*, both isolated from the same deep-tissue specimen. Notably, both organisms were also recovered from an independent nasal swab specimen, with concordant antimicrobial susceptibility profiles. However, histopathological examination did not reveal intracellular structures attributable to M. morganii, complicating assessment of its pathogenic role. Antimicrobial susceptibility testing, performed using VITEK 2 and interpreted according to EUCAST criteria, showed susceptibility of *K. pneumoniae* to standard therapeutic agents. In contrast, *M. morganii* exhibited resistance to aminopenicillins and amoxicillin–clavulanate while remaining susceptible to piperacillin–tazobactam, consistent with its known intrinsic AmpC β-lactamase profile (**Table 1**). Minimum inhibitory concentration (MIC) values for all tested agents are reported in **Table 1**. Before microbiological diagnosis, no systemic empirical antibiotic therapy had been administered. The patient had received only topical symptomatic treatment, resulting in partial improvement of epistaxis but persistent nasal obstruction. Following histopathological and microbiological confirmation, targeted antimicrobial therapy with oral levofloxacin (750 mg once daily) was initiated, with a planned treatment duration of 2–3 months. Considering the susceptibility profile of both isolates, the possibility of prolonged outpatient treatment, and the absence of systemic signs of infection, oral levofloxacin was selected. Unfortunately, the patient was subsequently lost to follow-up, and objective post-treatment clinical and endoscopic outcome data were not available.

**Table 1:** Antimicrobial susceptibility testing results for isolated pathogens.

Antimicrobial agent	*K. pneumoniae* subsp. *rhinoscleromatis* (MIC, µg/mL/SIR)	*M. morganii* (MIC, µg/mL/SIR)
Amoxicillin/clavulanate	≤4/S	>32/R
Piperacillin/tazobactam	≤4/S	≤4/S
Ceftazidime	≤0.12/S	8/R
Cefepime	≤0.12/S	0.25/S
Meropenem	≤0.25/S	≤0.25/S
Ciprofloxacin	≤0.06/S	≤0.06/S
Gentamicin	≤1/S	≤1/S
Amikacin	≤1/S	4/S
Trimethoprim/sulfamethoxazole	NT	NT
Colistin	NT	Intrinsic resistance

NT, not tested. Antimicrobial susceptibility testing was interpreted according to EUCAST 2025 criteria. Colistin results should be interpreted with caution according to EUCAST recommendations.

## Discussion

Rhinoscleroma is classically described as a chronic granulomatous infection caused by *K. pneumoniae* subsp. *rhinoscleromatis*. Although uncommon in high-income countries, it remains diagnostically challenging because of its indolent course and tumor-like presentation, frequently mimicking neoplastic processes. Definitive diagnosis relies on the identification of Mikulicz cells in conjunction with microbiological findings, although culture positivity is not universal. The traditional view of rhinoscleroma as a strictly monomicrobial disease largely reflects earlier microbiological approaches based on superficial sampling and conventional culture techniques. In the present case, deep-tissue biopsy enabled both histopathological confirmation and recovery of *K. pneumoniae* (consistent with subsp. *rhinoscleromati*s) together with *M. morganii*. Importantly, both or0ganisms were consistently isolated from two independent specimens (deep-tissue biopsy and nasal swab), with overlapping antimicrobial susceptibility profiles. This concordance reduces the likelihood of simple laboratory contamination and suggests a stable microbial association, although a definitive distinction between true co-infection and opportunistic colonization cannot be established in a single-case observation. Furthermore, histopathological examination demonstrated only the classical features of rhinoscleroma and did not reveal findings specifically attributable to *M. morganii*, supporting a cautious interpretation of its pathogenic role. Recent studies investigating chronic rhinosinusitis and other chronic inflammatory diseases of the upper airway have demonstrated that these conditions are frequently associated with complex microbial communities rather than single pathogens. Culture-independent approaches have revealed alterations in sinonasal microbial diversity, dysbiosis, and shifts in bacterial community structure, suggesting that chronic mucosal inflammation may facilitate the coexistence of multiple bacterial species (Abreu et al., 2012; Paramasivan et al., 2020; Fischer and Lee, 2024). Although rhinoscleroma remains a distinct granulomatous disease with a well-established etiological agent, these observations support the possibility that microbial complexity within chronically inflamed upper airway tissues may be underrecognized, particularly when microbiological investigations rely on superficial sampling or limited culture approaches. *M. morganii* is most commonly associated with urinary tract, intra-abdominal, and healthcare-associated infections; however, sporadic reports have documented its recovery from sinonasal and head-and-neck infections, indicating that its presence within inflamed upper airway tissues is biologically plausible (Liu et al., 2016; Alelyani et al., 2022). The chronic inflammatory microenvironment characteristic of rhinoscleroma, together with local tissue remodeling and disruption of mucosal barriers, may theoretically facilitate the persistence or coexistence of opportunistic Gram-negative organisms. Nevertheless, the pathogenic contribution of *M. morganii* to the disease process remains hypothetical and cannot be established from the present observation alone. Histopathological findings were entirely consistent with classical rhinoscleroma and do not provide evidence that *M. morganii* directly contributed to granuloma formation or disease progression. To our knowledge, published rhinoscleroma literature contains very limited information regarding systematically investigated bacterial co-isolates. Most reports focus on histopathological confirmation and recovery of *K. pneumoniae* subsp. *rhinoscleromatis*, whereas detailed microbiological characterization beyond the primary pathogen is rarely described (Mukara et al., 2014; Umphress and Raparia, 2018). The rarity of the disease and the historical reliance on superficial sampling may have contributed to underrecognition of potential microbial complexity. Therefore, while our findings should not be interpreted as evidence that rhinoscleroma is routinely polymicrobial, they suggest that comprehensive microbiological evaluation may occasionally reveal additional organisms of potential clinical relevance. Although the direct pathogenic role of *M. morganii* remains uncertain, its recovery had practical therapeutic implications in the present case. Specifically, the isolate exhibited resistance to aminopenicillins and amoxicillin–clavulanate while remaining susceptible to piperacillin–tazobactam, consistent with its known chromosomal AmpC β-lactamase expression (Jacoby, 2009; Tamma et al., 2019). Recognition of this resistance profile contributed to antimicrobial decision-making and highlights the value of performing susceptibility testing even in infections traditionally associated with a single pathogen. These findings have several clinical implications. First, rhinoscleroma should not automatically be assumed to be microbiologically simple when evaluated using deep-tissue sampling strategies. Second, reliance on superficial specimens may underestimate microbial diversity in chronic upper airway infections. Third, identification of co-isolated organisms with intrinsic resistance mechanisms may influence antimicrobial selection and stewardship decisions, even when their precise pathogenic contribution remains uncertain. This study has limitations. The absence of molecular confirmation limits subspecies-level certainty for *K. pneumoniae* identification. In addition, quantitative cultures and repeated deep-tissue sampling were not performed, precluding definitive assessment of the relative contribution of *M. morganii* to disease pathogenesis. A major limitation is the patient’s loss to follow-up after treatment initiation, preventing the evaluation of the clinical and endoscopic response. Prospective studies with structured follow-up will be necessary to clarify the impact of the treatment on disease resolution. Finally, the observational nature of a single case limits generalizability. Despite these limitations, this report supports the hypothesis that comprehensive microbiological evaluation, including deep-tissue and multi-sample analysis, may reveal previously unrecognized microbial complexity in infections traditionally considered monomicrobial. Further studies are needed to determine the prevalence and clinical significance of such findings in rhinoscleroma and other chronic granulomatous diseases of the upper airway.

## Conclusion

This case suggests that additional bacterial species may occasionally be recovered during comprehensive microbiological evaluation of rhinoscleroma. Whether such organisms represent true copathogens or secondary colonizers requires further investigation. Systematic deep-tissue sampling and microbiologically guided therapy remain essential for accurate diagnosis and optimized antimicrobial stewardship in chronic upper airway infections.

## Data Availability

The raw data supporting the conclusions of this article will be made available by the authors, without undue reservation.
